# Racial Misclassification of American Indian and Alaska Native People in the Electronic Medical Record: An Unexpected Hurdle in a Retrospective Medical Record Cohort Study

**DOI:** 10.2196/73086

**Published:** 2025-07-30

**Authors:** Ann Marie Rusk, Alanna M Chamberlain, Jamie Felzer, Yvonne Bui, Christi A Patten, Christopher C Destephano, Matthew A Rank, Roberto P Benzo, Cassie C Kennedy

**Affiliations:** 1 Mayo Clinic in Arizona, 13400 E. Shea Blvd, Scottsdale, AZ, 85259, United States; 2 Mayo Clinic, Rochester, MN, United States; 3 Emory University, Atlanta, GA, United States; 4 Mayo Clinic in Florida, Jacksonville, FL, United States

**Keywords:** electronic health records, EHR, health care disparities, Indigenous health, American Indian or Alaska Native, social determinants of health

## Abstract

Electronic health record data represent a rich data source; however, data accuracy must be considered prior to reporting health outcomes among American Indian and Alaska Native people. Using a hybrid approach to harmonizing data from multiple sources represents a valid method of assessing data integrity in this population.

## Introduction

Indigenous North Americans (American Indian and Alaska Native [AI/AN] people) in the United States have the shortest life expectancy among all racial or ethnic groups [1]. Disparate health and survival outcomes are influenced by social determinants of health (SDOHs)—factors that influence birth, health, life, and death—including health behaviors and systemic factors (eg, health care access) [2]. Addressing health care disparities requires equitable representation in public health data. In a retrospective cohort study that examined longitudinal cigarette smoking behaviors of Indigenous people in Olmsted County, Minnesota—a county without access to Indian Health Service clinics or hospitals—the magnitude of racial misclassification in electronic health record (EHR) data became an unexpected hurdle for the study team [3]. Most AI/AN people reside in urban areas or off reservation lands [4]. Understanding this population’s health behaviors is critical to informing interventions. Herein, we describe methods for harmonizing race data from multiple record sources to assure this frequently underrepresented and mischaracterized population’s accurate representation.

## Methods

Individuals with vital records (birth or death certificate) or EHR data (provider histories, EHR flowsheets, self-reports, or nursing documentation) indicating AI/AN race were identified in a longitudinal cohort study (2006‐2019) to assess smoking behaviors and pharmaceutical cessation aid uptake by race, sex, age, and indexed SDOHs [3]. Inclusion criteria were AI/AN race and availability of ≥1 year of smoking data. Exclusion criteria included non-AI/AN race and no smoking data available from 2006 to 2019. Patients were identified in the Rochester Epidemiology Project—a medical-record linkage system (established since 1966) inclusive of multiple health care delivery systems and population data for 99.9% of Olmsted County residents [5,6]. AMR, JF, and YB conducted data cleaning for all available records to resolve discordant records of AI/AN race; this included manual review of narrative EHR data, exclusion of individuals who used foreign language translation services, and review of patients’ vital records and records of parents and offspring. A sex- and age-matched (±5 years) non-AI/AN cohort was compared to the study cohort.

## Results

**Figure 1.**
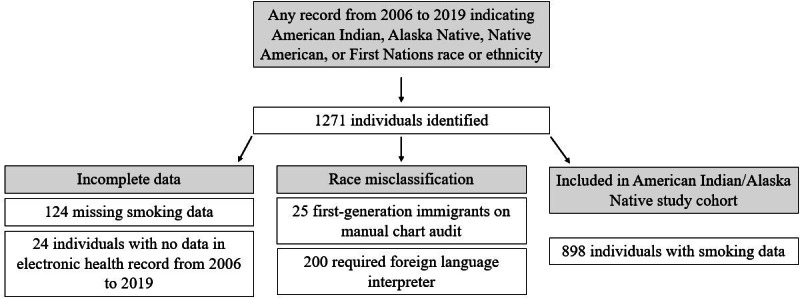
Inclusion and exclusion criteria applied to identify a cohort of American Indian or Alaska Native individuals in the Rochester Epidemiology Project from 2006 to 2019.

**Figure 2.**
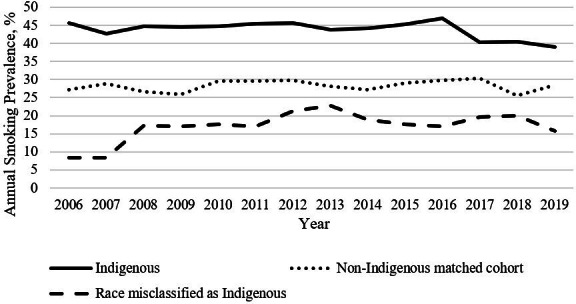
Annual smoking prevalence.

In total, 1271 individuals with ≥1 record indicating AI/AN race were identified; 148 were excluded (missing smoking data: n=124; no 2006-2019 EHR data: n=24). Manual review of the AI/AN cohort’s race and ethnicity data revealed 25 individuals who reported immigration from a non–North American country, and 200 individuals required foreign language interpreters for languages originating outside of North America (primarily languages originating from the Indian subcontinent and Southeast Asia). Final data cleaning resulted in a cohort of 898 AI/AN patients, demonstrating 17.7% (225/1271) racial misclassification [3] (Figure 1). The annual smoking prevalence for race-misclassified individuals (n=225) ranged between 8% and 23%; that for the AI/AN cohort (n=898) ranged between 39% and 47% (Figure 2). The matched cohort included 1780 individuals (White: n=1483, 83.3%; Black/African American: n=105, 5.9%; Asian: n=96, 5.4%; Hawaiian/Pacific Islander: n=4, 0.2%; other: n=68, 3.8%; declined: n=8, 0.4%; unknown: n=16, 0.9%) [3].

## Discussion

Harmonization of vital records and multiple EHR sources proved essential, as the magnitude of race misclassification (17.7%) in this study was higher than that in other AI/AN population studies, including a review of mortality data among AI/AN people in Washington State (12%) [7]. Without cohort validation, this study’s smoking prevalence would have been falsely lower due to lower smoking prevalence among race-misclassified individuals. Smoking behavior misrepresentation in medical literature would further exacerbate health care disparities in this underrepresented population. The Centers for Medicare & Medicaid Services have recognized the need to standardize data entry, releasing resources for health care organizations to improve demographic accuracy [8]. Until standardized data entry is implemented, additional methods for validating historical race data are necessary [9]. Data linkage—the harmonization of an individual’s data across different sources—represents a valid methodology [10]. Using a hybrid approach to AI/AN cohort validation—manual review of narrative documentation, vital records, and EHR input across multiple health systems—represents a potential method for smaller epidemiological studies. This study’s limitations included the inability to link data with tribal registries or Indian Health Service data and the time required to manually review records. Our methods may be used for counties where AI/AN individuals lack access to tribal health facilities (eg, Olmsted County). Besides manual data review, studies including AI/AN people should be conducted in concert with AI/AN people and tribes. This study was designed and conducted with oversight by an AI/AN community advisory board that expressed the critical importance of accurate race data. Studies using EHR data inclusive of AI/AN people should include measures for ensuring accurate race data and representation.

## Abbreviations

AI/AN: American Indian and Alaska Native
EHR: electronic health record
SDOH: social determinant of health
